# Reduced Offspring Viability Is Associated With Long‐Term Stability of a Narrow Avian Hybrid Zone

**DOI:** 10.1002/ece3.72120

**Published:** 2025-09-11

**Authors:** Kira M. Long, Michael J. Braun, Adolfo Muñoz Abrego, Ovidio Jaramillo, Todd M. Jones, Jeffrey D. Brawn

**Affiliations:** ^1^ Program in Ecology, Evolution and Conservation Biology University of Illinois Urbana‐Champaign Urbana Illinois USA; ^2^ Center for Conservation Genomics Smithsonian National Zoo and Conservation Biology Institute Washington DC USA; ^3^ Department of Vertebrate Zoology National Museum of Natural History, Smithsonian Institution Washington DC USA; ^4^ Department of Biology and Biological Sciences Graduate Program University of Maryland College Park Maryland USA; ^5^ Nance de Risco Bocas del Toro Republic of Panama; ^6^ Panama City Panama Republic of Panama; ^7^ Archbold Biological Station Venus Florida USA; ^8^ Department of Natural Resources & Environmental Sciences University of Illinois Urbana‐Champaign Urbana Illinois USA

**Keywords:** fitness, hatchability, hybridization, parasitism, reproduction, survival, tropical birds, viability

## Abstract

Fitness of hybrid individuals can shape the dynamics of hybrid zones and offer insight into speciation processes. Yet, accounts of hybrid fitness in natural hybrid zones are few, especially from tropical regions where species diversity is high and speciation processes could contrast with those at higher latitudes. We investigated a hybrid zone between the white‐collared manakin ( *Manacus candei* ) and the golden‐collared manakin ( *M. vitellinus* ), two lek‐breeding species characteristic of lowland forest habitat in Central America. Despite evidence of asymmetrical introgression and selection on male secondary sexual traits, ongoing sampling indicates that this hybrid zone is spatially stable with narrow clines, thus implying selection against hybrids. To evaluate hybrid viability, we estimated two components of hybrid fitness: survival of adults and egg hatching rates, and a possible selective pressure: prevalence of parasitism by vector‐borne haemosporidian parasites. Estimated survival was similar between parental and hybrid populations, and the prevalence of infections by *Plasmodium* spp. or *Haemoproteus* spp. parasites was uniformly low (4 positive cases, *N* = 268). Estimated rates of hatching success, however, were lower in nests from our hybrid population (one or two eggs failed to hatch in 70% of nests (*N* = 10)) compared to nests of the parental species ( *M. candei* 28.6% (*N* = 21) and *M. vitellinus* 19.0% (*N* = 7)). Thus, despite extensive admixture and clear evidence of introgression of male plumage traits under sexual selection, partial infertility or elevated rates of developmental mortality in hybrid offspring may underlie long‐term stability in this hybrid zone.

## Graphical abstract

We investigated hybrid viability in a tropical, avian hybrid zone governed by strong selection on male secondary sexual traits between the white‐collared manakin ( *Manacus candei* ) and golden‐collared manakin ( *M. vitellinus* ). We assessed putative hybrid fitness by investigating adult survival and hatching success along with a prospective selective pressure of parasitism by vector‐borne haemosporidian parasites. We found that hybrid populations display reduced hatching success, but no evidence of reduced survival or susceptibility to haemosporidian infections.

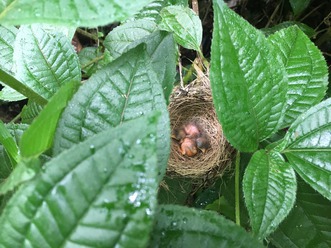

## RESUMEN

La aptitud de los individuos híbridos puede afectar la dinámica de las zonas híbridas y proveer información sobre los procesos de especiación. Sin embargo, los estudios de aptitud en zonas híbridas naturales son escasos, particularmente en regiones tropicales donde la biodiversidad es alta y los procesos de selección y especiación pueden diferir con respecto a los de latitudes más altas. Investigamos una zona híbrida entre el saltarín cuelliblanco ( *Manacus candei* ) y el saltarín cuellidorado ( *M. vitellinus* ), dos especies que se reproducen en leks, y distribuidas en bosques de tierras bajas de Centroamérica. A pesar de que hay evidencia de introgresión asimétrica y selección en los caracteres sexuales secundarios masculinos, estudios recientes indican que esta zona híbrida es espacialmente estable y con clinas estrechas, lo que implica selección en contra de individuos híbridos. Para evaluar la viabilidad de saltarines híbridos, estimamos dos componentes de la aptitud híbrida: la supervivencia en adultos y las tasas de eclosión de los huevos, y una posible presión selectiva: la prevalencia de parásitos hemosporidios transmitidos por vectores. La supervivencia de adultos fue similar entre las poblaciones parentales e híbridas, y la prevalencia de infecciones parasíticas por *Plasmodium* spp. o *Haemoproteus* spp. fue uniformemente baja (4 casos positivos, *N* = 268). Sin embargo, las tasas estimadas de eclosión fueron menores en los nidos de una población híbrida (uno o dos huevos no eclosionaron en el 70% de los nidos; *N* = 10) en comparación con los nidos de las especies parentales ( *M. candei* 28.6% (*N* = 21) y *M. vitellinus* 19.0% (*N* = 7)). Por lo tanto, a pesar de la extensa mezcla genética y la clara evidencia de introgresión de los rasgos del plumaje masculino debido a selección sexual, la infertilidad parcial o las elevadas tasas de mortalidad durante el desarrollo en poblaciones híbridas podrían ser la causa de la estabilidad a largo plazo en esta zona híbrida.

## 1 Introduction

Hybridization is a key evolutionary event that invites inquiry into the processes that generate and maintain species diversity. Hybrid zones form at the overlapping edges of two or more species' ranges and are dynamic systems that can move across a landscape or remain spatially stable. If hybrids have a selective advantage, they are expected to expand into the ranges of one or both parental species (Buggs 2007). Alternatively, hybrid zones can remain narrow and spatially stable if hybrids have low fitness compared to the parental species (Barton and Hewitt 1985). Thus, hybrid fitness can be key to understanding hybridization dynamics and the processes underlying speciation. Yet, accounts of hybrid fitness in natural hybrid zones are few, especially from tropical regions where species diversity is high and speciation rates and processes could contrast with those at higher latitudes. Tropical regions have been found to have slower rates of secondary sympatry and longer divergence times between sister taxa than temperate species of mammals and birds; these differences likely stem from a variety of factors such as latitudinal differences in the strength of barriers to range expansions, competitive exclusion, and rate of reproductive isolation from premating barriers (Weir and Price 2011; Weir and Schluter 2007).

Reduced viability of hybrids and consequent fitness costs can be realized through a variety of mechanisms across vertebrate taxa, including reduced survival rates and fertility in fish (Gilk et al. 2004; Muhlfeld et al. 2009; Stelkens et al. 2015) and mammals (Adavoudi and Pilot 2021; Lancaster et al. 2007; White et al. 2012). For birds, higher rates of hatching failure have been reported in both outbred and inbred populations (Ålund et al. 2023; Bronson et al. 2005; Maxwell et al. 2021; Neubauer et al. 2014; Sætre et al. 1999), suggesting that reduced hatching success could be an important indicator of underlying genetic incompatibilities. Furthermore, mating systems that result in highly skewed reproductive success, such as lek‐breeding systems, invite exploration of the tension between sexual selection and natural selection in hybrid zones and possible effects on hybrid fitness.

**FIGURE 1:**
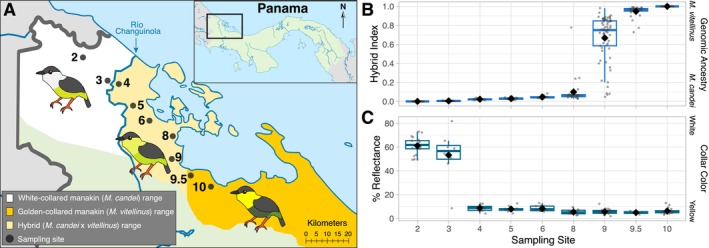
The *Manacus* hybrid zone. (A) Study region in Bocas del Toro, Panama. The black dots represent the sampling sites and are numbered according to Brumfield et al. (2001) and Long et al. (2024). Sampling site 2 is parental *M. candei* , site 10 is parental *M. vitellinus* , and site 9 is the genomic center of the hybrid zone with the most highly admixed hybrid individuals. Note that the phenotypic transition where yellow collar plumage switches to white collar plumage occurs at the Río Changuinola between sites 3 and 4. The map has been reproduced from Long et al. (2024). (B) Genomic hybrid index derived from 10,000 SNPs for 318 manakins from across the *Manacus* hybrid zone sequenced in Long et al. (2024) (i.e., the “contemporary” dataset sampled in 2017–2020). A hybrid index of 0 indicates complete *M. candei* ancestry (e.g., site 2) and a hybrid index of 1 indicates complete *M. vitellinus* ancestry (e.g., site 10). The mean hybrid index at each site is denoted by a diamond. This boxplot figure has been modified from Long et al. (2024). (C) Collar color of male *Manacus* plumage from across the hybrid zone as % reflectance at 490 nm. Higher reflectance values denote white plumage and lower values denote yellow. The mean % reflectance at each site is denoted by a diamond. This boxplot figure has been made using data from Long et al. (2024).

We assessed the viability of hybrids from two species of lek‐breeding birds, the golden‐collared manakin ( *Manacus vitellinus* ) and the white‐collared manakin ( *Manacus candei* ), which hybridize in western Panama. Male manakins perform elaborate mating displays for females seeking mates (Day et al. 2021; Kirwan and Green 2011), with most copulations going to few males (Emlen and Oring 1977; Lill 1974; Payne 1984). In this hybrid zone, female *Manacus* prefer males with yellow collar plumage (Stein and Uy 2006), resulting in the observed introgression of yellow *M. vitellinus* plumage traits for more than 50 km beyond the genomic transition from *vitellinus* to *candei* (Brumfield et al. 2001; Parsons et al. 1993) (Figure 1A–C). Despite multiple lines of evidence that sexual selection favors males with yellow collars (McDonald et al. 2001; Stein and Uy 2006), the genomic transition between the two parental species has remained spatially stable and narrow for at least 30 years, while the darker olive belly color of *M. vitellinus* has continued to introgress into populations that are genomically like *M. candei* , indicating a decoupling of sexual and natural selection (Long et al. 2024).

The spatial stability of this hybrid zone within a narrow genomic transition despite strong sexual selection and ongoing introgression of male secondary sexual traits suggests the influence of selective pressures against hybrids, specifically, reduced fitness owing to lower adult survival, reduced reproductive output, or both. The specific selective pressure(s) causing reduced hybrid fitness could be direct effects leading to hybrid sterility (White et al. 2012) and offspring inviability (Bolnick and Near 2005), more subtle factors such as susceptibility to disease and parasites (Goldberg et al. 2005), or physiological impairments like increased oxidative stress (Barreto and Burton 2013; Du et al. 2017; Petrović et al. 2023), which, for male manakins, could compromise their performance in a highly competitive lek‐breeding system (Chen et al. 2025).

Parasitism from vector‐borne blood parasites can be a strong selective force in wild bird populations (Atkinson 2023; Fecchio et al. 2019; Lachish et al. 2011; LaPointe et al. 2012). Hybrids could be especially disadvantaged if they inherit susceptibility to parasites from both parental species, leading to increased selective pressure on hybrids (Derothe et al. 2001; Krasnovyd et al. 2017). In manakins, there is evidence of reduced fitness from avian malarial infections causing impaired mating behavior on the lek, such as decreased vocalization and display rates (Bosholn et al. 2016). Furthermore, the polygynous lek‐breeding system in manakins may increase exposure to parasites and their vectors when individuals gather for breeding displays (Fecchio et al. 2011; Tella 2002). Thus, given strong selection on male traits, the structure of lek‐breeding systems, and the expectation that a significant parasite load will negatively impact male secondary sexual traits (Hamilton and Zuk 1982), parasites could be an important selective pressure compromising hybrid fitness in the *Manacus* hybrid system.

To assess whether hybrids have reduced fitness in the *Manacus* hybrid zone, we estimated and compared survival rates of adults and hatching rates of eggs (proxies of hybrid fitness) between hybrids and both parental species. Given the apparent genomic stability of the *Manacus* hybrid zone, we predicted that (1) adults in the genomic center of the hybrid zone would experience reduced survival rates and/or (2) hatching success would be reduced in nests at the genomic transition compared to nests from the parental species populations. We also estimated the prevalence of haemosporidian infection (i.e., avian malarial and malarial‐like vector‐borne blood parasites *Plasmodium* spp. and *Haemoproteus* spp.) as a possible selective pressure on *Manacus* hybrids. We predicted that hybrid *Manacus* individuals would have a greater prevalence of blood parasites than the parental species.

## 2 Methods

### 2.1 Study Species and Sampling Sites

*Manacus vitellinus* and *Manacus candei* (Pipridae) are frugivorous, neotropical birds characteristic of secondary or edge habitats in lowland humid forests. *M. vitellinus* occurs in Panama and western Colombia, while *M. candei* occurs from the Yucatan to Panama. These species' ranges overlap in Bocas del Toro, Panama, where they are known to hybridize. *Manacus* males congregate at leks where they perform elaborate courtship displays for visiting females (Day et al. 2021; Kirwan and Green 2011; Lill 1974). After mating, only females provide parental care. Both species and their hybrids build open cup nests. As is typical for neotropical birds, *M. candei* and *M. vitellinus* lay 1–2 eggs per nest (Chapman 1935; Stutchbury and Morton 2023). The number of nesting attempts per year has not been formally assessed, but multiple renesting attempts are common in neotropical birds (Stutchbury and Morton 2023). Likewise, the average lifespan of the parental species has not been empirically evaluated; however, modeling of longevity and generation times for *M. vitellinus* and *M. candei* using life history traits has estimated a maximum longevity of 9.48 years and a generation length of 2.46 years for *M. vitellinus* and 9.95 years maximum longevity and 2.53 years generation length for *M. candei* (Bird et al. 2020). The estimated species divergence time between *M. candei* and *M. vitellinus* is estimated to be 970 kya (using a demographic modeling method; (Lim et al. 2024)) and 1.15 mya (using a phylogenetic method; (Harvey et al. 2020; Kumar et al. 2017)), making the hybridizing *Manacus* manakins relatively newly diverged (the average time to speciation in birds is generally 1.5 million years in temperate birds and 2 million years in birds at the equator), and thus unlikely to have already evolved complete hybrid infertility or inviability (Price 2008; Price and Bouvier 2002; Weir and Schluter 2007).

**FIGURE A1:**
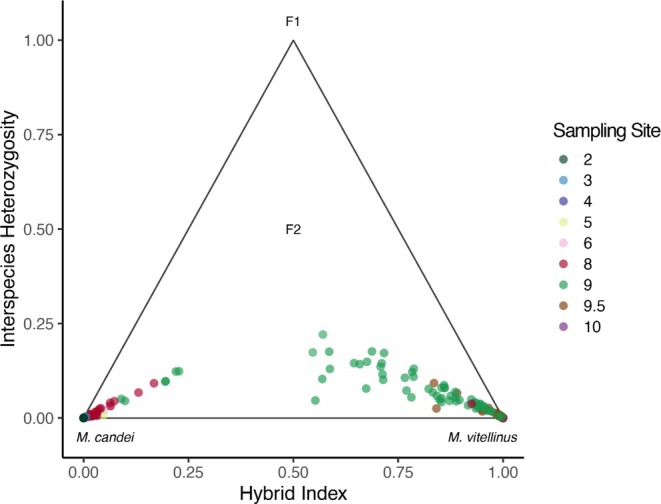
Hybrid assessment via the relationship between the genomic hybrid index (0 designates *M. candei* , and 1 designates *M. vitellinus* ) and interspecies heterozygosity (proportion of sites with alleles derived from both parental populations) calculated using 2000 diagnostic SNPs for 318 manakins from across the *Manacus* hybrid (i.e., the “contemporary” dataset of Long et al. (2024)). Colors denote the nine sampling sites across the *Manacus* hybrid zone. The location of the F1 and F2 hybrids would be notated on the triangle. None of the sequenced individuals shows evidence of being early‐generation hybrids. This triangle plot figure has been modified from Long et al. (2024).

We sampled at nine sites in a transect across Bocas del Toro, Panama (Figure 1A), many of which were assessed previously by Brumfield et al. (2001), Yuri et al. (2009), Parchman et al. (2013), and Long et al. (2024). We used the same numbering scheme as those studies to maintain consistency in the ordinal designations of the sampling sites. Note that sampling site 9.5 was a new addition to the transect, added by Long et al. (2024) to increase spatial resolution between sites 9 and 10, with the convention of a decimal place used to preserve the numerical ordering of the sites while keeping the original site numbers the same. Sites 2 and 10 represent the focal parental species sites for parental *M. candei* and *M. vitellinus* , respectively. Sites 3 and 9.5 represent additional parental sites, and all other sites correspond to populations with various levels of parental admixture that we refer to as the “hybrid populations”. Site 9, in particular, represents the hybrid center and is the genomic transition between the two parental populations, displaying the largest variation in parental ancestry (Figure 1B), hereafter referred to as the hybrid focal population (Brumfield et al. 2001; Long et al. 2024). We did not find evidence of “pure” parental species individuals present at the focal hybrid site, but rather all previously genotyped individuals appear to be late‐generation hybrids, displaying some level of admixture (Figures 1B and A1, Long et al. 2024). Logistics restricted us to sampling at focal sites 2, 9, and 10 for estimating annual survival and hatching success, while all 9 sampling sites were assessed for prevalence of haemosporidian infections.

### 2.2 Capture, Mark, and Release of Individuals

Birds were captured with mist nets in 2017–2020 from late February to June. We set up mist nets (36‐mm mesh “ATX” type) on leks every 3 days near the displaying males' courtship arenas to capture displaying males, floating juveniles, and visiting females. Nets were checked every 30 min from 0800 to 1700 h, and all captured manakins were given an aluminum band with a unique identification number and a combination of plastic colored leg bands for individual identification after release (Raw capture record: Table S1). All protocols involving the capture and handling of live birds were reviewed and approved by the Illinois Animal Care and Use Committee and the Smithsonian Tropical Research Institute Animal Care and Use Committee (Illinois IACUC numbers: 15234 & 18239; STRI ACUC numbers: 2016‐0301‐2019 & 2019‐0115‐2022).

### 2.3 Sex Determination

Sex and reproductive status were determined in the field using definitive adult male plumage or the presence of a brood patch (only females incubate). However, immature males and non‐breeding females look nearly identical, with overall olive plumage and yellow bellies, thus their sex was confirmed at a later time using an avian polymerase chain reaction (PCR) molecular sexing protocol that targets an intron of the *CHD1* gene on the Z and W chromosomes (Fridolfsson and Ellegren 1999). Briefly, we extracted blood samples of < 100 μL/bird by puncturing the brachial vein. Blood was stored in Longmire's lysis buffer (Longmire et al. 1997) and kept frozen at −20°C until laboratory analysis. We used primers 2550F and 2718R (2550F = 5′‐GTT ACT GAT TCG TCT ACG AGA‐3′; 2718R = 5′‐ATT GAA ATG ATC CAG TGC TTG‐3′) in a master mix with TaKaRa Ex Taq DNA Polymerase reagents (Takara Bio USA Inc) to perform the PCR outlined in Table A1. The PCR products were visualized for scoring after electrophoresis on a 2% agarose gel stained with GelRed Nucleic Acid Gel Stain (Biotium, Fremont, CA, USA). The targeted Z chromosome region was a 600 bp fragment that would be present in both sexes, while only females had a second 450 bp amplified fragment from the W chromosome (Molecular sexing results: Table S2).

### 2.4 Estimating Adult Survival

We rotated mist netting at the three focal sites each day so that each site was sampled twice a week for the entire five‐month field season, resulting in similar sampling effort at each focal site for each sampling year. In 2020, however, we were unable to continue mist netting operations for the entire 5‐month field season due to the COVID‐19 pandemic, and thus all mark‐recapture data from 2020 were dropped from subsequent analyses because sampling effort was too low. We calculated apparent survival using Cormack‐Jolly‐Seber (CJS) models for open populations using *Program MARK* (White and Burnham 1999). CJS models calculate apparent survival as the product of the probability of true survival and fidelity to the study area (Lebreton et al. 1992). Since mortality and emigration cannot be distinguished, apparent survival is an underestimate of true survival (Schaub and Royle 2014). For each individual, we created an encounter history using a custom python script (https://github.com/kiralong/manacus_HZ_fitness_ms/tree/main/Apparent_survival/MARK_extract_input_file). Briefly, this script takes a list of each date a bird was captured, the sampling site it was captured at, its unique band ID, and field estimated sex and/or molecular sexing assay result to create a year‐by‐year capture record for that individual and exports an appropriately formatted input file (.inp) for *Program MARK*. For example, if a bird was captured in 2017, not recaptured in 2018, but recaught in 2019, it would be denoted as ‘101’ for the 2017, 2018, and 2019 field seasons, respectively. The script will also check all instances of sex identification for an individual from the field and assign a single, final sex determination (i.e., check if the individual was assigned the same sex at every capture and cross‐reference with molecular sex data), which is helpful for identifying juvenile males that gain adult male plumage at a later date.

*Program MARK* then provides estimates of apparent survival (denoted as φ) and accounts for possible heterogeneity in recapture probability (i.e., probability of recapturing an individual given presence in the sampling area, denoted as *p*) among groups. We examined whether adult survival varied by hybrid status by running a set of a priori models with hybrid status modeled as a group. We had three groups: white‐collared manakins (parental sampling site 2), golden‐collared manakins (parental sampling site 10), and hybrid individuals at the genomic center of the hybrid zone (hybrid sampling site 9). The “hybrid group” included all marked individuals from sampling site 9, where birds display a wide range of mixed ancestry (Figures 1B and A1). While assessing the impact of various levels of mixed ancestry on survival is of interest, genomic data was not available for all recaptured individuals, and limiting the *MARK* analysis to only individuals with a known hybrid index (as determined in Long et al. (2024)) resulted in unreliable models, likely due to reduced sample size.

We also accounted for potential differences in survival between male and female manakins by incorporating sex as a covariate. Specifically, we ran a set of models with no effect of hybrid status (i.e., groups combined) or by group, and with and without the effect of year. After running these initial models, we then controlled for sex by adding it as a covariate to our top model; as survival varied by year in our top model, we constructed two separate models, one with sex modeled as constant (i.e., same effect of sex across years) or by year (effect of sex varies by year). We determined our top model using Akaike's information criteria, adjusting for small sample size (AICc; Burnham and Anderson 2002) and used our top model to determine whether adult survival varied by hybrid status. We also examined whether the 95% confidence intervals overlapped based on hybrid status using the top model, including a group effect (averaged across years if there was a year effect). We determined whether there was an effect of sex on survival based on the estimate and 95% confidence intervals of the top model, including the effect of sex. Finally, to check if recapture probability differed between the sexes, we ran a second analysis with groups for all hybrid‐sex combinations (i.e., parent species 1‐males, parent species 1‐females, hybrid‐males, hybrid‐females, etc.). We constructed a model where we pooled groups of the same sex (i.e., all male groups, all female groups) and compared the 95% confidence intervals to determine if recapture probability differed between the sexes.

### 2.5 Hatching Success

We systematically searched for nests in the understory at leks and the surrounding forest every 3 days. We also used radio telemetry to locate nests by fitting females with transmitters (PicoPip Ag376 Tags from Lotek Wireless Inc., Canada). The transmitters weighed < 5% of the birds' body weight (as recommended by Brander and Cochran 1969; Naef‐Daenzer et al. 2001; Barron et al. 2010) and were attached using the Rappole harness technique (Rappole and Tipton 1991). At each located active nest, we “candled” eggs with a flashlight to inspect the interior of the egg every 3 days to track egg development (Birkhead et al. 2008). Briefly, eggs candled right after being laid will glow yellow when illuminated and can have an observably distinct yolk. If an egg has been fertilized, it will further develop red vasculature, and the egg will glow red when illuminated. As the egg is incubated, a developing embryo can be observed, usually appearing as a small, dark shadow that grows until completely eclipsing the interior of the egg, except for the air cell, which also grows larger and becomes increasingly slanted as incubation continues up until hatching. If a female was found currently sitting on the nest at a three‐day nest check, we did not flush the female and skipped candling the eggs that day to reduce the likelihood of nest abandonment. Given that fertilized eggs can fail within the first few hours after fertilization and would appear nearly identical in phenotype to truly unfertilized eggs when using a field candling method, we focus our analyses on eggs that hatched or failed to hatch by the end of the incubation period. To accurately determine if an egg failed due to fertilization failure versus early embryo mortality would require microscopy of egg contents (Assersohn et al. 2021). Nests that failed due to extrinsic factors such as predation, flooding, or human activity such as logging or herding cattle were not included in our analyses. Thus, we defined an egg as failing to hatch based on the definition of hatching failure provided by Marshall et al. 2023; that is, the nest was not abandoned or destroyed by extrinsic factors such as predation, accident, or extreme weather, and the egg failed to hatch by the end of the expected incubation period (18 days for *M. vitellinus* (Brawn et al. 2011), and we assumed a similar incubation period for *M. candei* or hybrids). To our knowledge, all eggs in our analyses were incubated (Hatching data: Table S3). For nests at the focal hybrid site (site 9), the exact level of admixture for each parent is unknown, as we do not have genotype information for every parent of every nest; however, all adults that were sequenced previously from the hybrid focal site displayed some level of admixture between *M. candei* or *M. vitellinus* (Long et al. 2024).

To compare hatching rates between the focal parental and hybrid sampling sites, we performed two statistical tests. First, we calculated the proportion of eggs that failed to hatch relative to all eggs that were laid in each focal group and then performed a two‐tailed Fisher's Exact Test for count data using the Freeman–Halton extension to test a 2 × 3 contingency table to compare the three groups using fisher.test() in base R (R Core Team 2022). Second, to account for the non‐independence of eggs within the same nest, we fit the hatching success data to a binomial regression, using success/failure to hatch for each egg within a single nest to fit a generalized linear model with a binomial distribution. We used the glm() function with the binomial family available in base R, and structured our data so that each row is a nest, with response columns for the success/failure of each egg in the nest (R Core Team 2022). This method counts a binomial ‘success or failure’ outcome for one or more eggs within a nest and thus avoids non‐independence of eggs within a single nest.

### 2.6 Haemosporidian Assays

We tested for avian blood parasites in 268 individuals (Malaria data: Table S4). DNA was extracted from whole blood using an Autogen Genprep 965, using the ‘Animal Tissue’ protocol, which involves Proteinase K digestion, phenol extraction, and alcohol precipitation. The quantity of DNA was determined using a Qubit BR dsDNA assay (Life Technologies), as described in Long et al. (2024). Each sample was screened at least twice to reduce false positives in the dataset. We screened each blood sample for the presence or absence of the vector‐borne avian malaria parasites and malaria‐like parasites from the genera *Plasmodium* or *Haemoproteus*, respectively; hereafter, haemosporidia is used to refer to both assayed genera. We used PCR to target a highly conserved region of the parasites' mitochondrial DNA, the 154‐bp *16S rRNA* coding sequence. This PCR test will result in a positive result for the presence of either parasitic lineage, and does not differentiate if the infection is *Plasmodium* spp. or *Haemoproteus* spp. We used the primers *343F* and *496R* from Fallon et al. (2003) with the following sequences: *343F* = 5′‐GCT CAC GCA TCG CTT CT‐3′ and *496R* = 5′‐GAC CGG TCA TTT TCT TTG‐3′ to perform the PCR outlined in Table A2. The PCR products were visualized for scoring after electrophoresis on a 2% agarose gel stained with GelRed Nucleic Acid Gel Stain (Biotium, Fremont, CA, USA). Samples were always run with two positive controls to corroborate positive results, and two negative controls to detect any possible contamination, but no contamination was found in any PCR run. All samples were then screened a second time with the same protocol to reduce false positive results. Samples that were positive in the screening PCR were then advanced to a second nested PCR assay that targets the *cytochrome b* gene in the parasites' mitochondria. For this nested PCR assay, we used the *HAEM* primers from Waldenstrom et al. (2002): *HAEMNF* = 5′‐CAT ATA TTA AGA GAA TTA TGG AG‐3′, *HAEMNR2* = 5′‐AGA GGT GTA GCA TAT CTA TCT AC‐3′, *HAEMF* = 5′‐ATG GTG CTT TCG ATA TAT GCA TG‐3′, and *HAEMR2* = 5′‐GCA TTA TCT GGA TGT GAT AAT GGT‐3′ to perform the PCR outlined in Table A3. Samples that were positive in all three PCR assays (twice in the screening step and once in the Waldenstrom step, for three PCR tests total) were considered positive in the final dataset.

## 3 Results

### 3.1 Hatching Success

**FIGURE 2:**
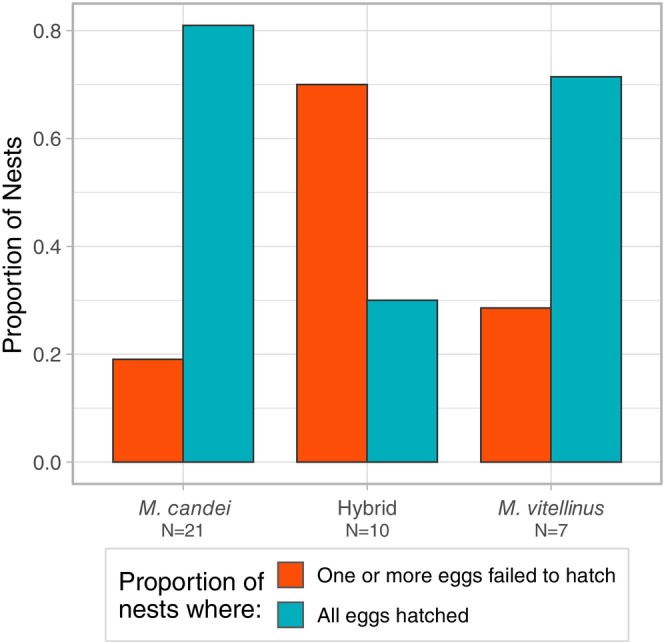
Paired bar plots of the proportion of nests in which: All eggs hatched successfully in a nest (the blue bar on the right of each focal group) or one or both eggs failed to hatch within a nest (the red bar on the left of each focal group) across the three focal *Manacus* populations in the hybrid zone. Proportions are derived from *N* = 21 *M. candei* , 10 hybrid, and 7 *M. vitellinus* nests. Both bars together represent all monitored nests within a focal group.

We located 47 active nests across the three focal sampling sites. Nine (19%) nesting attempts failed prior to hatching (6 *M. candei* , 3 hybrid, and 0 *M. vitellinus* ). Of the remaining 38 nests, we monitored the developmental progress of eggs throughout the incubation period in 21 ( *M. candei* ), 10 (hybrid), and 7 ( *M. vitellinus* ) nests. All nests contained 1 or 2 eggs at all three focal sites. Six of the 38 nests (16%) contained only one egg for the entire incubation period (4 *M. candei* , 1 hybrid, and 1 *M. vitellinus* ). Of all the nests with singleton eggs, 2 (33%) failed to hatch. One or both eggs failed to hatch in 11 of 32 (34%) nests with two eggs. Overall, the proportion of failed eggs from the total eggs laid within each focal site was 13% ( *M. candei* , 5 of 38), 42% (hybrid, 8 of 19), and 23% ( *M. vitellinus* , 3 of 13) (Fisher's Exact Test, *p*‐value = 0.0473). Grouping hatching failure by nest to account for the non‐independence of eggs, 70% of nests from the hybrid focal population had one or both eggs fail to hatch, a proportion significantly greater than *M. vitellinus* (28.6%) or *M. candei* (19.0%) (Figure 2). (GLM Binomial, Wald statistic *z* value = −2.348, df = 37, SE = 0.67, *p*‐value = 0.0189).

### 3.2 Egg Candling Observations of Failed Eggs

Across all three focal sites, 13 of the 16 eggs that failed to hatch never developed red vasculature (4 *M. candei* , 6 hybrid, 3 *M. vitellinus* ) (Hatching Data: Table S3). Two of the failed eggs developed the characteristic red veining (1 *M. candei* , 1 hybrid), and the *M. candei* egg further developed a small embryo before ceasing development. For one egg from the hybrid population, we were unable to get candling observations during incubation because the female was on the nest during every 3‐day nest check; however, when checking the nest after the 18‐day incubation period, we found one nestling in the nest, and the other egg was missing. We believe the female removed the egg after it failed to hatch. We observed this egg removal behavior once more in the hybrid focal population and once in *M. candei* . All three removals occurred in nests where two eggs were laid, and the failed egg disappeared after the successful egg hatched.

### 3.3 Adult Survival

**FIGURE 3:**
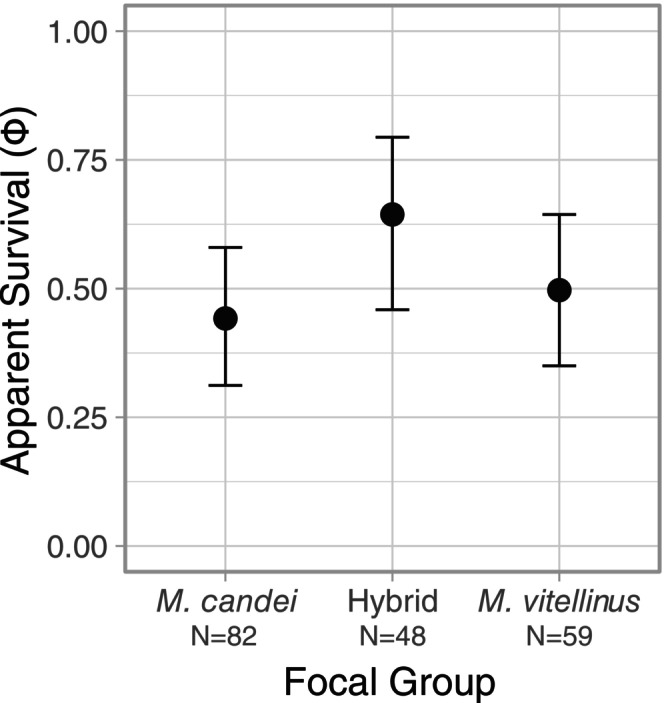
Estimated annual survival of hybrids and two parental species of *Manacus* manakins with 95% confidence intervals. Estimates based on 189 individuals from *M. candei* (*N* = 82), *M. vitellinus* (*N* = 59), and genomic center hybrids (*N* = 48) from 2017 to 2019.

**FIGURE A2:**
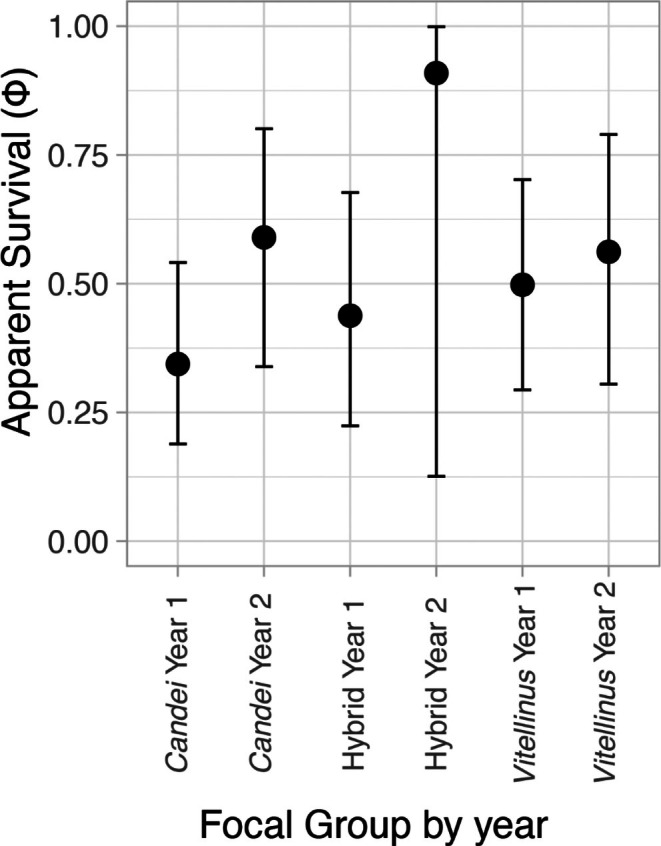
Estimated annual survival by year for three focal groups of *Manacus* manakins with 95% confidence intervals. Estimates based on 189 individuals from *M. candei* (*N* = 82), *M. vitellinus* (*N* = 59), and genomic center hybrids (*N* = 48). Year 1 is the 2017 to 2018 survival estimate, and year 2 is the 2018–2019 survival estimate.

We captured 316 individuals from *M. candei* (*N* = 131), *M. vitellinus* (*N* = 65), and genomic center hybrids (*N* = 120), consisting of adult males (*N* = 111), breeding females (*N* = 102), and immature males, immature females, or non‐breeding females of unknown age (*N* = 103) from February through June in the 2017, 2018, and 2019 sampling seasons. However, of these birds, only a subset had a chance to be resighted in a subsequent year (i.e., only birds first captured in 2017 or 2018). Thus, birds included in *Program MARK* analyses were 189 individuals from *M. candei* (*N* = 42 males, *N* = 40 females, 1.05:1 M:F), *M. vitellinus* (*N* = 35 males, *N* = 24 females, 1.45:1 M:F), and genomic center hybrids (*N* = 28 males, *N* = 20 females, 1.40:1 M:F). The overall top model (model 1) did not include the effect of group nor the effect of sex, suggesting that there were no differences in survival by hybrid status or sex (Table 1). Based on the top model that included the effect of group (averaged across years; model 5), apparent survival rates of hybrids (φ = 0.64, SE 0.089) and parental species ( *M. candei* φ = 0.44, SE 0.070, *M. vitellinus* φ = 0.50, SE 0.077) displayed overlapping 95% confidence intervals (Figure 3), and we found no significant effect of sex for the top model that included sex as a covariate (β = 0.10, 95% CI −0.587 to 0.788; model 2). Thus, we have little evidence that survival differs for hybrid individuals. Notably, adult survival appeared to vary by year, but this was largely due to a high estimate of survival from 2018 to 2019 for hybrids, though this estimate is quite uncertain given the large 95% confidence intervals (95% CI 0.126–0.999) (Figure A2). The recapture probability for manakins across the hybrid zone was 0.68 (SE 0.101). The recapture probability for males was 0.83 (SE 0.13, CI 0.44–0.96) and females was 0.65 (SE 0.11, CI 0.40–0.83), but since the confidence intervals overlap, we do not have evidence that recapture probability differs significantly by sex.

**TABLE 1:** Results from Cormack–Jolly–Seber (CJS) models examining variation in survival by hybrid status, time (year), and sex across a white‐collared and golden‐collared manakin hybrid zone in Bocas del Toro, Panama, 2017–2019.

Model number	Model	ΔAICc	*w*_*i*_	*k*	Deviance
1	φ(time), *p*(constant)	0.000	0.469	3	321.419
2	φ(time + sex[constant]), *p*(constant)	1.994	0.173	4	321.337
3	φ(time + group), *p*(constant)	2.069	0.167	7	315.065
4	φ(time + sex[time]) *p*(constant)	4.085	0.061	5	321.333
5	φ(constant + group) *p*(constant)	4.210	0.057	4	323.553
6	φ(constant) *p*(constant)	4.386	0.052	2	327.862
7	φ(time + group) *p*(constant + group)	6.288	0.020	9	314.950

### 3.4 Haemosporidian Parasitism

We screened 268 individuals for haemosporidian parasites (*Plasmodium* spp. and *Haemoproteus* spp.). Only 4 individuals tested positive for an active infection in all three PCR tests (Table 2). All infected individuals were *M. candei* (three adult males and one adult female) from the same sampling site (site 2, 8.33%, *N* = 48). Of all *M. candei* individuals tested (sites 2 and 3, *N* = 73), 5.48% had active haemosporidian infections. No hybrid (*N* = 132) or *M. vitellinus* (*N* = 63) individuals tested positive for haemosporidian infection. Overall, from the 268 individuals assessed from across the hybrid zone, only 1.49% tested positive for a haemosporidian infection.

**TABLE 2:** Sampling of *Manacus* individuals screened for haemosporidians and the number positive per sampling site.

Sampling site	Hybrid status	Individuals screened (*n*)	Number positive	Percent infected (%)
2	*M. candei*	48	4	8.33
3	*M. candei*	25	0	0.0
*M. candei* subtotal		73	4	5.48
4	Hybrid	24	0	0.0
5	Hybrid	20	0	0.0
6	Hybrid	20	0	0.0
8	Hybrid	21	0	0.0
9	Hybrid	47	0	0.0
Hybrid subtotal		132	0	0.0
9.5	*M. vitellinus*	30	0	0.0
10	*M. vitellinus*	33	0	0.0
*M. vitellinus* subtotal		63	0	0.0
Total		268	4	1.49

*Note:* The four positive individuals were all *M. candei* and composed of 3 adult males and 1 adult female.

## 4 Discussion

Our findings suggest that reduced hatching success, but not reduced adult survival, is a consequence of hybridization in the *Manacus* hybrid zone. The focal population at the genomic center of the hybrid zone had significantly lower hatching success and a larger proportion of nests containing failed eggs when compared to the parental species. Estimated adult survival rates were similar among focal populations, and this did not differ significantly by sex. Additionally, we found the prevalence of infection by *Plasmodium* spp. and/or *Haemoproteus* spp. parasites was uniformly low across the entire hybrid zone. We posit that reduced reproductive success is the main avenue contributing to the many stable, narrow clines observed in the genomic center of the *Manacus* hybrid zone (Long et al. 2024). We acknowledge that our sample sizes are relatively small for some analyses, despite thousands of person‐hours expended in collecting the data, and thus some of our findings should be interpreted with caution since small sample sizes are more prone to spurious statistical significance than larger datasets. Despite this, we believe that these results advance our understanding of the dynamics of this hybrid zone.

### 4.1 Reduced Reproductive Success Signaling Genetic Incompatibilities

Reduced hatching success in nests from the hybrid population was the clearest evidence we found of a fitness cost to hybridization. Reduced hatching success (or nesting/fledging success as related proxies of reproductive success) has been observed in other hybrid zones and taxa (Ålund et al. 2023; Kruuk et al. 1999; Muñoz et al. 2010; Stelkens et al. 2015; Svedin et al. 2008), but is not universal (Casas et al. 2012; Megna et al. 2014; Vallender et al. 2007; Walsh et al. 2016). For the parental species, we found 13% ( *M. candei* ) and 23% ( *M. vitellinus* ) rates of hatching failure, generally in line with the mean estimated rates in birds of 16.79% (Marshall et al. 2023). The proportion of hatching failure for nests in the hybrid focal population, however, was 42%, significantly higher than the parental species.

In birds, higher rates of hatching failure often appear in small populations where inbreeding is likely (Assersohn et al. 2021; Marshall et al. 2023; Westemeier et al. 1998). Marshall et al. 2023 found the highest rates of hatching failure in captive birds classified as threatened (by the IUCN Red List classifications), with 42.84% hatching failure. Threatened taxa are likely to be characterized by populations that are small, bottlenecked, or otherwise lacking genetic diversity. Inbreeding depression has been highlighted as one of the major drivers of hatching failure in threatened bird species (reviewed in Assersohn et al. 2021); however, we lack equivalent, systematic assessments of outbreeding depression/hybridization on hatching success. Inbreeding depression is generally due to decreasing heterozygosity, thereby increasing the likelihood of deleterious alleles coming to fixation at homozygous sites. Conversely, outbreeding depression is more likely due to breaking associations of coadapted alleles at multiple loci across the genome (Shields 1982). While inbreeding and outbreeding depression are seemingly opposites, the shared phenotype of hatching failure invites inquiry into whether inbreeding and outbreeding are tapping into similar developmental processes and if additional studies of hybrid hatching failure could help elucidate common drivers of hatching failure.

The stage of development at which an embryo dies can offer clues as to which biological processes are going awry and guide further investigations. Embryo mortality in birds, in general, occurs in either the early or late stages of development before hatching (Assersohn et al. 2021; Romanoff 1949). Early embryo mortality (e.g., < 72 h after successful fertilization), in particular, is commonly associated with genetic problems (Assersohn et al. 2021; Shook et al. 1971). Experimental work in inter‐genic hybrids between chickens (*Gallus gallus domesticus*) and Japanese quail ( *Coturnix japonica* ), which have extremely high hatching failure in F1 hybrids and 100% hybrid male sterility, found that 75%–80% of hybrid embryos ceased development by the second day of incubation (Ishishita et al. 2016). Failure in these first two incubation days means that embryos stopped developing before the formation of discernible embryonic structures, including extraembryonic membrane and blood island formation (Ishishita et al. 2016). Thus, these sorts of common early embryonic egg failures may be indistinguishable from unfertilized eggs when using the field candling techniques we employed.

While we were unable to predictably monitor *Manacus* eggs from their first few hours of incubation, all hybrid eggs that failed to hatch by the end of the incubation period did not form an embryo large enough to detect with our field candling (i.e., they all retained a clearly defined yellow yolk or had bright red vascularization when illuminated), suggesting that either fertilization failure occurred or embryo development was arrested in the early stages when the embryo was very small (within the first 2 days). However, all but a single *M. candei* egg from the parental species also failed to develop a visible embryo, although there were fewer failed eggs to observe in the parental species. Nevertheless, without using microscopy to examine the eggs that failed to hatch, we cannot determine if these eggs failed due to fertilization failure or early embryonic death, which would each point to different underlying mechanisms.

For example, if hybrid eggs truly have a higher fertilization failure, this could imply the underlying mechanism is related to deficiencies in hybrid sperm, such as low quantities or poor sperm function. However, if hybrid eggs were failing from early embryonic death in the first few days of incubation, this would point to lethal problems in developmental processes such as protein synthesis, cell proliferation, and gastrulation, which all occur in intergenic chicken‐quail hybrids from misregulated gene expression in these same pathways (Ishishita et al. 2020). Future studies should candle eggs to monitor egg development, collect failed eggs to determine the exact cause of hatching failure, and/or artificially incubate eggs to detail embryo development in a controlled environment to determine exactly when and how hybrid embryos cease development. This would also present an opportunity to investigate Haldane's Rule in the unhatched eggs by using a molecular sexing method on the failed eggs to determine if hybrids of the heterogametic sex are more likely to fail to hatch (Cowell 2023; Haldane 1922; Ishishita et al. 2016). Artificial incubation has been successfully done in manakins (Jones and DuVal 2019) and could be an important tool for future investigations of hybrid hatching success to alleviate the pressure of high predation rates on the nests of interest. Additionally, assessing hybrid fertility by investigating hybrid male sperm counts is another avenue by which hybrid sterility or fertilization failure could be assessed.

Genetic incompatibilities between the two parental species, for example, Bateson–Dobzhansky–Muller incompatibilities (Bateson 1909; Coyne and Orr 2004; Dobzhansky 1936), are a likely mechanism underlying the reduced hatching success in the *Manacus* hybrid zone. The genetic center of the hybrid zone may be acting as a filter (Martinsen et al. 2001), taking out inviable embryos early on in development and leaving only hybrid adults with the successful allelic combinations. We observed similar rates of adult apparent survival between the hybrid and parental focal groups, consistent with this idea. We find further evidence from Vernasco et al. (2024), who observed that birds sampled at site 9 have longer telomeres and increased heterozygosity in comparison to parental populations at sites 2 and 10. While the specific fitness effects of telomere lengths remain unknown in *Manacus*, longer telomere lengths are often associated with higher fitness, providing preliminary support for hybrid manakins having equal‐to‐higher fitness than the parents once individuals with incompatible alleles are removed during development. Other avenues should be explored as well to assess additional selective pressures on hybrid populations, such as metabolic or physiological robustness in hybrid individuals that could affect their courtship displays or dispersal ability. Moreover, collecting failed hybrid eggs is desirable for genetic studies to uncover unsuccessful allelic combinations seen only in unhatched hybrids. Such genes are likely vitally important for the speciation process (Presgraves 2010).

Reduced hatching success could be a particularly strong selective pressure for hybrid zone dynamics in tropical species, given generally small clutch sizes (1–2 eggs) and high rates of nesting failure (Brawn et al. 2011; Stutchbury and Morton 2023). If, for example, a temperate bird lays a 5–8 egg clutch and half fail to hatch from hybrid infertility or inviability, then that nest could still produce 3–4 successful offspring (as seen in Bronson et al. 2005). In contrast, many tropical birds, including manakins, lay only two eggs, which would result in only one successful offspring if half fail to hatch. While tropical species tend to renest more often due to longer breeding seasons (Stutchbury and Morton 2023), higher hatching failure from genetic incompatibilities would likely still be present in subsequent renesting attempts. Thus, hybrid hatching success could be a key factor in regulating avian hybrid zone dynamics in the tropics, and more studies on tropical hybrid zones could confirm if this is a common mechanism across taxa and latitudes.

### 4.2 Haemosporidia as a Selective Pressure in Hybrid Zones and Manakins

Parasitism has been proposed as an important mechanism for shaping hybrid zones (reviewed in Theodosopoulos et al. 2019); however, there are mixed results on the effects of avian blood parasites on hybridization dynamics (Cozzarolo et al. 2018; Reullier et al. 2008; Rice et al. 2021; Roth et al. 2021). We found only 1.49% positive infections for haemosporidian parasites across the entire *Manacus* hybrid zone in western Panama, indicating that *Plasmodium* and *Haemoproteus* do not play a major role in the hybridization dynamics of our focal system. This result is somewhat surprising, given the ubiquitous prevalence of haemosporidian parasites in other neotropical species such as clay‐colored thrush ( *Turdus grayi* ) (Ricklefs and Sheldon 2007) and bananaquit ( *Coereba flaveola* ) (Antonides et al. 2019; Ricklefs et al. 2011), both of which are commonly found adjacent to *Manacus* leks.

In other manakin species, the prevalence of haemosporidian infections tends to be low compared to the rest of the avian community (when surveyed), but there are mixed results depending on the species assessed. For example, blue‐crowned manakins ( *Lepidothrix coronata* ) had 35% prevalence (Bosholn et al. 2016), while the wire‐tailed manakins ( *Pipra filicauda* ) had only 5.6%, the lowest prevalence in an assemblage of 39 species (Svensson‐Coelho et al. 2013). Across 30 species of manakins surveyed by Fecchio et al. (2017), only 22 had any malaria detected, with an average prevalence of only 10.9% (ranging from 0% to 50%), and *Manacus* spp. maintained some of the lowest malaria prevalence, with *Manacus vitellinus* at 7.5% and *Manacus manacus* at only 6.7%. Other studies on *Manacus* manakins have all found low levels of haemosporidian infection, including zero infections detected in *M. candei* (Valkiūnas et al. 2004) and 2 out of 6 infected *M. vitellinus* individuals (Ricklefs and Sheldon 2007), but sample sizes to date have been quite small. Our larger sampling from *M. candei* and *M. vitellinus* does, however, corroborate these previous findings that *Manacus* have low haemosporidian prevalence.

We hypothesize that manakins, especially *Manacus*, might not readily contract haemosporidian infections. However, we did not collect blood samples from non‐target species on the leks, so we cannot say for certain if *Manacus* manakins specifically have abnormally low prevalence rates compared to the local avian communities around secondary forests in Bocas del Toro, Panama. There has been one previous study surveying avian blood parasites in the region of the *Manacus* hybrid zone (Galindo and Sousa 1966), which found 25.5% of 249 species in the region around Almirante had evidence of blood parasites, and *Manacus* had 2 out of 36 individuals positive for blood parasite infection. Based on the survey area of the study, these *Manacus* were likely hybrid individuals. Nevertheless, the contemporary status of haemosporidians in Bocas del Toro is unknown, including whether there are fewer vectors to aid in the spread of haemosporidian parasites in the region, fewer species/genera of parasites, or fewer infected hosts due to increased parasite resistance, etc. Future studies of blood parasite infections at the community level will clarify whether low prevalence is systemic among species in the region's bird communities or specific to *Manacus* manakins.

## 5 Conclusion

We explored hybrid viability in a tropical, avian hybrid zone governed by strong selection on male secondary sexual traits. We assessed putative hybrid fitness by investigating adult apparent survival and hatching success along with a prospective selective pressure, blood‐borne parasitism by haemosporidians. We found that hybrids have reduced hatching success, but no evidence of reduced survival or increased susceptibility to avian malarial infections. Thus, we believe that hybrid dysfunction is manifesting through reduced reproductive success and could be a key factor contributing to hybrid zone stability. Linking reduced hatching success to specific genetic incompatibilities in hybrid genomes is an important next step to uncovering the dynamics of hybridization and speciation.

## Author Contributions

**Kira M. Long:** conceptualization (equal), data curation (lead), formal analysis (lead), funding acquisition (supporting), investigation (lead), methodology (lead), project administration (equal), software (lead), supervision (equal), validation (lead), visualization (lead), writing – original draft (lead), writing – review and editing (equal). **Michael J. Braun:** conceptualization (equal), funding acquisition (equal), project administration (equal), supervision (equal), writing – review and editing (equal). **Adolfo Muñoz Abrego:** investigation (equal), project administration (supporting). **Ovidio Jaramillo:** investigation (equal), project administration (supporting), writing – review and editing (supporting). **Todd M. Jones:** formal analysis (supporting), investigation (supporting), writing – review and editing (supporting). **Jeffrey D. Brawn:** conceptualization (equal), funding acquisition (lead), methodology (supporting), project administration (equal), resources (lead), supervision (equal), validation (equal), writing – review and editing (equal).

## Conflicts of Interest

The authors declare no conflicts of interest.

## Supporting information


**Data S1‐S4:** ece372120‐sup‐0001‐DataS1‐S4.xlsx.

## Data Availability

Scripts for analyses and data processing are available at https://github.com/kiralong/manacus_HZ_fitness_ms. The data used to generate the results in this study are provided in the supplementary Excel file (“Supplementary_Data.xlxs: S1.Raw_capture_record, S2. Molecular_sexing_results, S3.Hatching_data, and S4.Malaria_data”) associated with this manuscript.

## Appendix A

**TABLE A1:** Molecular sexing protocol.

Master mix (TaKaRa reagents)		PCR regime		
Reagent	Proportion of volume in a 10 μL reaction	Temperature (Celsius)	Time (MM:SS)	Cycles
Water	5.635	94	2:00	1
10× Buffer (Mg^2+^ free)	1.5	94	0:45	31
dNTPs (2.5 mM)	0.15	48	0:45	
MgCl_2_ (25 mM)	0.65	72	0:45	
2550F (10 μM)	0.5	72	10:00	1
2718R (10 μM)	0.5			
Taq (5 U/μL)	0.065			
DNA template	1.0			

*Note:* Specific methods for the master mix and PCR cycles to determine sex in birds. TaKaRa reagents are the standard kit concentrations from the TaKaRa Ex Taq (Mg2+ free Buffer) kit (Catalog # RR01AM). The PCR protocol and primers 2550F and 2718R are based on Fridolfsson and Ellegren (1999).

**TABLE A2:** PCR protocol for screening for avian blood parasites.

Master mix (TaKaRa reagents)		PCR regime		
Reagent	Proportion of volume in a 10 μL reaction	Temperature (Celsius)	Time (MM:SS)	Cycles
Water	5.95	94	2:00	1
10× Buffer (Mg^2+^ free)	1.0	94	0:50	35
dNTPs (2.5 mM)	1.0	55	0:50	
MgCl_2_ (25 mM)	0.6	72	0:25	
343F (20 μM)	0.2	72	2:00	1
496R (20 μM)	0.2			
Taq (5 U/μL)	0.05			
DNA template	1.0			

*Note:* TaKaRa reagents are the standard kit concentrations from the TaKaRa Ex Taq (Mg2+ free Buffer) kit (Catalog # RR01AM). The PCR protocol and primers *343F* and *496R* are based on Fallon et al. (2003).

**TABLE A3:** Waldenstrom nested PCR protocol to detect haemosporidians.

Master mix (TaKaRa reagents)		PCR regime		
Reagent	Proportion of volume in a 25 μL reaction	Temperature (Celsius)	Time (MM:SS)	Cycles
**Waldenstrom outer reaction**				
Water	15.625	94	3:00	1
10× buffer (Mg^2+^ free)	2.5	94	0:30	35
dNTPs (2.5 mM)	2.0	55	0:30	
MgCl_2_ (25 mM)	1.75	72	0:45	
HAEMNF (10 μM)	1.0	72	10:00	1
HAEMNR2 (20 μM)	1.0			
Taq (5 U/μL)	0.125			
DNA template	1.0			
**Waldenstrom inner reaction**				
Water	15.625	94	3:00	1
10× Buffer (Mg^2+^ free)	2.5	94	0:30	35
dNTPs (2.5 mM)	2.0	55	0:30	
MgCl_2_ (25 mM)	1.75	72	0:45	
HAEMF (10 μM)	1.0	72	10:00	1
HAEMR2 (20 μM)	1.0			
Taq (5 U/μL)	0.125			
Waldenstrom Outer Reaction Product	1.0			

*Note:* TaKaRa reagents are the standard kit concentrations from the TaKaRa Ex Taq (Mg2+ free Buffer) kit (Catalog # RR01AM). For this nested PCR assay, we used the *HAEM* primers from Waldenstrom et al. (2002).
